# Neoxanthin is undetectable in human blood after ingestion of fresh young spinach leaf

**DOI:** 10.1371/journal.pone.0288143

**Published:** 2023-07-19

**Authors:** Mihoko Sekiya, Shigenori Suzuki, Yusuke Ushida, Ikuo Sato, Hiroyuki Suganuma

**Affiliations:** 1 Innovation Division, Nature & Wellness Research Department, KAGOME CO., LTD., Tochigi, Japan; 2 International University of Health and Welfare Hospital, Tochigi, Japan; Poznan University of Life Sciences: Uniwersytet Przyrodniczy w Poznaniu, POLAND

## Abstract

In a previous study, we demonstrated that the carotenoid neoxanthin was contained in young leafy vegetables including spinach and showed a fat accumulation inhibitory effect *in vitro*. To evaluate the bioavailability of neoxanthin, a raw young spinach leaf (100 g day^–1^ for 4 weeks) intake test was performed on 14 participants (36.5 ± 8.0 years; male:female ratio = 9:5). Neoxanthin, neochrome, β–carotene, and lutein concentration in the spinach and blood of participants (before and after the test) was measured using high performance liquid chromatography. Neither neoxanthin nor neochrome was detected in the blood samples, whereas β–carotene and lutein concentration significantly increased (1.4– and 1.9–fold, respectively) during testing. Neoxanthin bioavailability in humans is low; thus, it is unlikely to have a fat accumulation inhibitory effect *in vivo*, contrary to the result *in vitro*. Ingesting the leafy vegetables raw can help maintain high neoxanthin levels, but it is not beneficial for neoxanthin bioavailability.

## Introduction

Obesity is a serious health issue in many countries and regions since it causes metabolic syndrome and leads to serious cardiovascular and neurological disorders [1, 2]. Obesity can be caused by genetic factors but lifestyle–related habits, especially the lack of exercise and excess energy (sugar and lipid) intake are also very impactful. Therefore, various approaches have been employed worldwide to prevent obesity, and many studies on food materials and ingredients that contribute to preventing obesity have been conducted. Some of them have been utilized as ingredients of food with functional claim after their efficacy in humans has been confirmed [3, 4]. The investigation of a wide range of studies on the relationship between food materials or ingredients and obesity prevention has revealed that there was a negative association between blood concentration of carotenoids, and the levels of metabolic syndrome–related markers, based on the results of multiple epidemiological studies [5–7]. We take most of carotenoids from vegetables and fruits. A review of the previous animal and cell studies on the fat accumulation inhibitory effects showed that xanthophyll carotenoids (e.g., β–cryptoxanthin and zeaxanthin) are more effective than carotene carotenoids (e.g., lycopene and β–carotene), and that even among the xanthophyll–based carotenoids, compounds like fucoxanthin (derived from seaweed) and neoxanthin [derived from leafy vegetables, such as spinach (*Spinacia oleracea*) and Japanese mustard spinach (*Brassica rapa var*. *perviridis*)] that contain an epoxide moiety show particularly remarkable effects [8–14].

Neoxanthin has been reported to be heat–unstable [15]. Therefore, in previous studies, we measured the neoxanthin contents and evaluated its action on the cells of young leaves of leafy vegetables that are generally eaten raw (which are customarily called young leaf [16, 17], and demonstrated that the young leaves of spinach, arugula, and beet are rich in neoxanthin and that these leaves are expected to suppress fat accumulation [18].

There is only a limited number of studies that have evaluated the absorption of neoxanthin by the body and its fat accumulation suppressing effect *in vivo*. According to a study by Asai *et al*. [19], orally–ingested neoxanthin is partially converted to neochrome via exposure to an acidic environment in the gastrointestinal tract, but it is detected in the blood of mice, together with neochrome. In a subsequent study by Asai *et al*. [20], neoxanthin and neochrome were detected in the blood of healthy human participants who had ingested sautéed spinach (mature leaves) containing neoxanthin for one week. However, since the amount of neoxanthin (or neochrome) detected in the blood was extremely low compared to the amount of β–carotene and lutein also contained in the sautéed spinach, the authors reported that the bioavailability of neoxanthin in humans was low, and the efficacy of neoxanthin *in vivo* has not been verified since then. According to a review on the absorption and metabolism of various carotenoids [21], the bioavailability of carotenoids differs greatly between animals and humans, and only a limited number of carotenoids have been identified in human tissues. The review discusses why the bioavailability of neoxanthin is lower in humans, but the reason is not clear.

In this study, to re–evaluate the bioavailability of neoxanthin using an evaluation system that can detect and measure neoxanthin more reliably, we carried out an intake test in human participants using the young spinach leaves (raw leaves) evaluated in the previous study [18].

## Material and methods

### Test food

This study used commercially available young spinach leaves [Young Spinach, Kajitsudo Co., Ltd. (Kumamoto, Japan)] as with a previous study [18]. The displayed nutritional contents of the young spinach leaves (100 g^–1^) were as follows: energy = 20 kcal; proteins = 3.2 g; fats = 0 g; carbohydrates = 3.2 g; sugars = 0.8 g; dietary fiber = 2.4 g; salt equivalent = 0.16 g, and iron = 2.8 mg. The content of various carotenoids (neoxanthin, β–carotene, and lutein) in young spinach leaves was measured using high performance liquid chromatography (HPLC) according to previous reports [22]. The neoxanthin, β–carotene, and lutein standard preparations used to prepare the calibration curve were purchased from FUJIFILM Wako Pure Chemical Co. (Osaka, Japan). The following three types of salad dressing were purchased from supermarkets to accompany the young spinach leaves when eating: Pietro Dressing (fats: 7.1 g/15 g, PIETRO Co., Ltd., Fukuoka, Japan), Kewpie Surioroshi Onion Dressing (fats: 2.7 g/15 g, Kewpie Co., Ltd., Tokyo, Japan), and Kewpie Tasty Dressing Japanese Flavored Onion (fats: 5.7 g/15 g, Kewpie Co., Ltd).

### Participants

The recruited participants were healthy men and women who were 20 years–old or older and who are working in the Innovation Division, KAGOME CO., LTD. (Tochigi, Japan). All participants provided written informed consent and the exclusion criteria were the following: 1) having a history of diabetes, digestive, circulatory, liver, kidney, or heart diseases; 2) being pregnant or breastfeeding, or planning on becoming pregnant in the near future; 3) being possibly allergic to the test food (young spinach); 4) being a smoker; 5) taking supplements that contain calcium at the time of the study; 6) having had calculi (stones) in the past or having been told by a physician that they are at risk of developing calculi; and 7) having been deemed ineligible to participate for other reasons at the discretion of the principal investigator or research director.

### Study design

A single–arm before–and–after study was conducted. During the 4 weeks (28 days) of the intervention period, participants were asked to ingest 100 g of young spinach leaves every day with an arbitrary amount of salad dressing. The participants were instructed to chew the leaves well when eating. The ingestion time was any time on an empty stomach (before or during meals) and was left unspecified. Blood samples were taken from participants on the day before and on the day of starting the food intake test, and post–intervention blood samples were taken on the day before or on the last day of food ingestion testing. Plasma samples were prepared from the collected blood, and these samples were kept at –80°C until the measurements.

The plan of this study was approved by the KAGOME CO., LTD. Research Ethics Committee (Reference No.: 2019–R14) before registration on the clinical trial registry system UMIN–CTR (UMIN000043060), and the study was conducted while complying with the “World Medical Association Declaration of Helsinki” and “Ethical Guidelines for Medical and Health Research involving Human Participants” (2014 Ministry of Education, Culture, Sports, Science and Technology/Ministry of Health, Labour and Welfare Notification No. 3).

### Measurement of the plasma neoxanthin and neochrome levels

The plasma neoxanthin and neochrome levels were measured using HPLC by referring to a previous report [19]. A volume of 800 μL of a dichloromethane:methanol = 2:1 (v/v) solution was added to 200 μL of plasma. The mixture was then stirred for 30 s and shaken for 15 min before recovering the supernatant in a spitz glass. The supernatant obtained by repeating the same operations with the remaining pellets was combined with the previously recovered supernatant, and the solvent was distilled off using nitrogen. A volume of 200 μL of a dichloromethane:methanol = 2:1 (v/v) solution was added to the spitz glass to completely dissolve the dried concentrate. After this, the sample was centrifuged (11,400 × *g*, 1 min), filtered through a 0.22–μm filter and injected into a HPLC system. The HPLC system (Shimadzu Corporation, Kyoto, Japan) consisted of an LC–30AD pump, a SIL–30AC auto–sampler, a TSK–gel ODS column (TSK–gel ODS 80Ts 2.0 mm × 250 mm, 5 μm, TOSOH, Kyoto, Japan), a YMC guard column (YMC Guard Cartridge 10 × 2.1 mm, S–5 μm, YMC, Kyoto, Japan), a CTO–20AC column oven (30°C) and a CBM–20A system controller. An SPD–M30A diode array was used for the detector, and the measurement wavelength was set to 450nm, which is suitable for carotenoids. An acetonitrile:methanol:water [75:15:10 (v/v/v) containing 0.1% ammonium acetate; Solvent A] solution and a methanol:ethyl acetate [70:30 (v/v); Solvent B] solution were used as the mobile phases for HPLC. The flow rate used was 0.3 mL min^-1^, and the following elution program was configured: 0 min to 25 min: Solvent A 100% to Solvent B 100% (linear gradient); 25 min to 28 min: Solvent B 100%; 28 min to 30 min: Solvent B 100% to Solvent A 100%; 30 min to 40 min: Solvent A 100%. Neoxanthin was quantified (μg mL of plasma) from the peak area of the HPLC chromatogram using a calibration curve prepared using a standard compound. As we could not obtain the standard substance for neochrome, we performed a before–and–after comparison of the peak area of the HPLC chromatogram that was estimated to correspond to neochrome, referring to the report by Asai *et al*. [19, 20].

### Measurement of the plasma β–carotene and lutein levels

The plasma β–carotene and lutein levels were measured using HPLC according to a previously reported method [6, 7]. A volume of 20 μL of a mixture of 8–apo–carotenal (Cosmo Bio Co., Ltd., Tokyo, Japan), δ–tocopherol (Mitsubishi Chemical CO., Ltd., Tokyo, Japan), and retinyl acetate (Merck Biopharma Co., Ltd., Tokyo, Japan), which was used as an internal standard, as well as 1.0 mL of an ethanol solution containing 0.01% (w/v) butylhydroxytoluene (BHT) were added to 200 μL of plasma. Furthermore, 4 mL of a hexane:dichloromethane = 4:1 (v/v) solution was added before stirring the mixture for 30 s. After centrifuging (1,710 × *g*, 5 min), the supernatant was recovered. The supernatant obtained by repeating the same operations with the remaining pellets was combined with the previously recovered supernatant, and the solvent was distilled off using nitrogen. Then, 200 μL of a HEAT (hexane/acetone/ethanol/toluene = 10/6/7/6):ethanol = 4:6 (v/v) solution was added to completely dissolve the dried matter, and the sample obtained was centrifuged (11,400 × *g*, 1 min) and injected into an HPLC system. The HPLC system used has been described previously, but the column used was a YMC carotenoid column (YMC Carotenoid 2.0 × 250 mm, 5 μm, YMC, Kyoto, Japan), and the detector (SPD–M20 diode array) was set to a wavelength of 450 nm. A methanol:methyl–tert–butyl ether:water [75:5:20 (v/v/v) containing 0.5% triethylamine and 0.01% BHT; Solvent A] solution and a methanol:methyl–tert–butyl ether:water [8:90:2 (v/v/v) containing 0.5% triethylamine and 0.01% BHT; Solvent B] solution were used as the mobile phases for HPLC. The flow rate used was 0.2 mL min^–1^, and the following elution program was configured: 0 min to 25 min: Solvent A 100% to Solvent B 100% (linear gradient); 25 min to 28 min: Solvent B 100%; 28 min to 30 min: Solvent B 100% to Solvent A 100%; 30 min to 40 min: Solvent A 100%. Β–carotene and lutein were quantified (μg mL^–1^ of plasma) based on the peak area of the HPLC chromatogram using the calibration curves prepared from respective standards.

### Statistical analysis

The blood carotenoid concentration data were presented as mean ± standard deviation (n = 14). The differences in the blood carotenoid concentration before and after the intervention period were analyzed by Student’s *t*–test, and the statistically significant differences were defined by a *P* < 0.05. Statistical analysis was conducted using the EZR software (ver. 1.37; Saitama Medical Center Jichi Medical University) [23].

## Results

### Carotenoid content in young spinach leaves

We measured the carotenoid content in the 9 lots of young spinach leaves that were used in this study. The mean neoxanthin content was 3.5 ± 0.1 mg 100 g –^1^ fresh weight (FW). This meant that the ingestion of 100 g of young spinach leaves per day provided more neoxanthin (3.5 mg day^–1^) than that provided in the study by Asai *et al*. [20] (3 mg day^–1^). The mean β–carotene and lutein contents were 3.2 ± 0.2 and 3.3 ± 0.1 mg 100 g –^1^ FW, respectively.

### Participant profile

**Fig 1:**
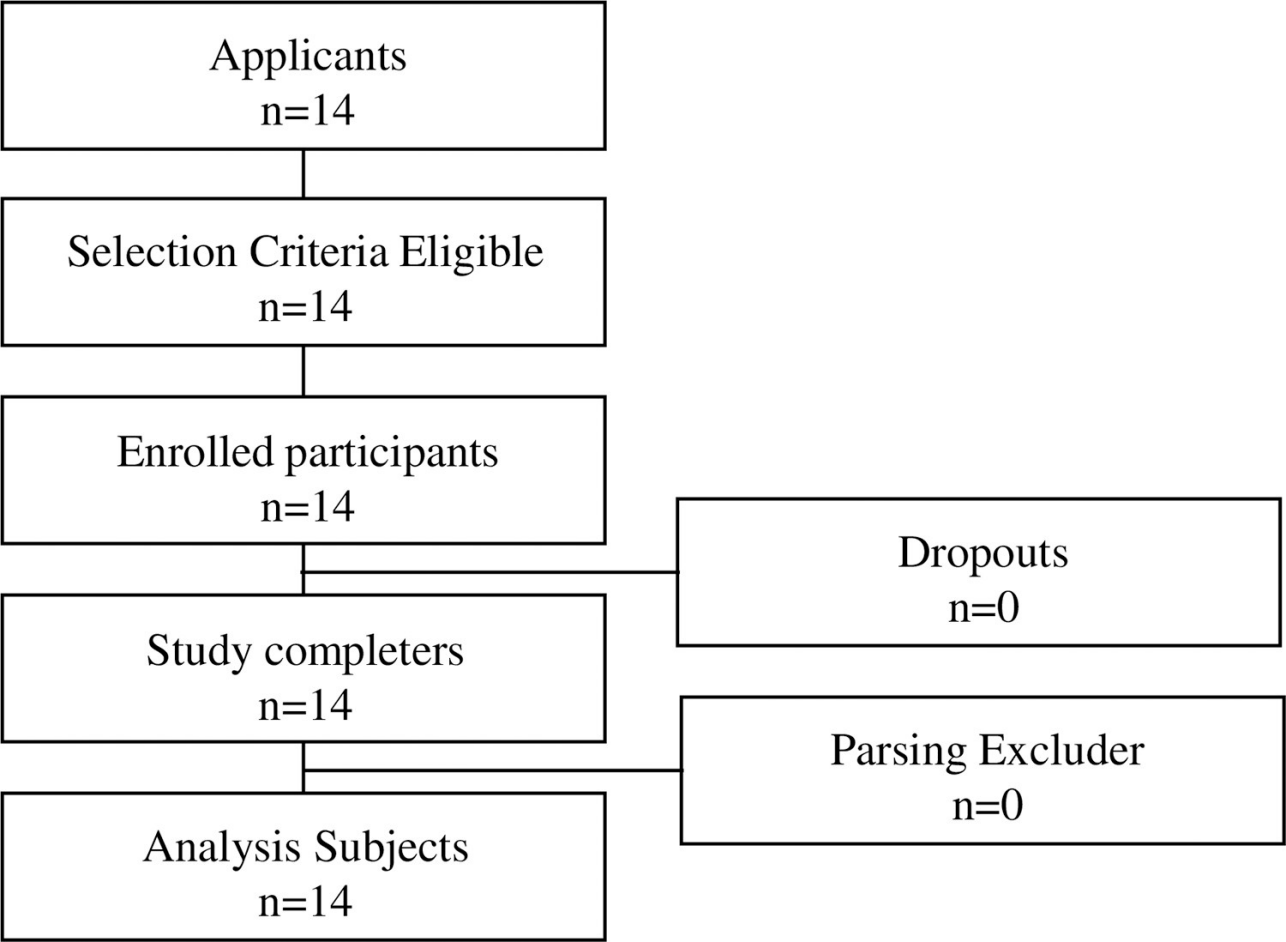
CONSORT flow diagram. A total of 14 participants were subjected to analysis.

A total of 14 participants that provided written consent were enrolled in this study. All the 14 participants completed the study while complying with the study rules and restrictions; thus, they were all analyzed (**Fig 1**). Basic analysis showed that the mean age of the 14 participants was 36.5 ± 8.0 years, the male/female ratio was 9:5, and the mean body mass index (BMI) was 22.1 ± 2.4 kg m^–2^. We did not observe any adverse events related to the study plan or test food throughout this study.

### Plasma neoxanthin and neochrome concentration

**Fig 2:**
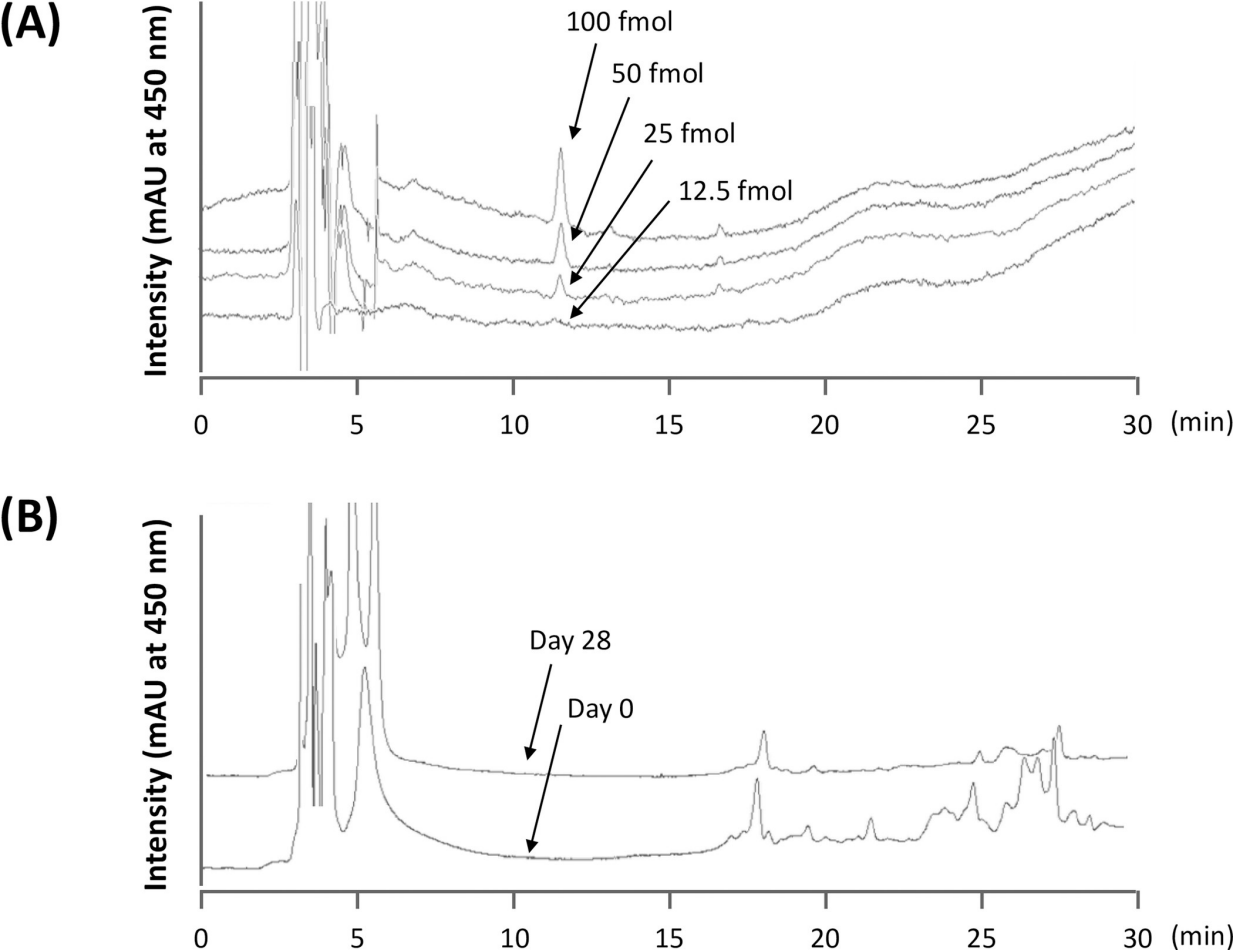
HPLC chromatograms of neoxanthin. (A) Peaks of neoxanthin standards (12.5, 25, 50, and 100 fmol/injection) were detected at 450 nm at around 12 min of retention time. (B) Representative HPLC chromatograms of plasma-derived samples from participants before (Day 0) and after (Day 28) intake period of young spinach leaf (100 g day^-1^). There were no peaks around 12 min of retention time.

We produced a measurement system for neoxanthin and neochrome by referring to the method by Asai *et al*. [19]. **Fig 2A** shows the chromatograms resulting from measuring the standard neoxanthin. A quantifiable signal–to–noise ratio was observed at the 25 fmol/injection level. In other words, we have produced a measurement system that can quantify the concentration of neoxanthin and neochrome at a limit of detection of 0.5 nmol L^–1^ of plasma when injecting 50 μL of a plasma–derived sample.

By measuring the neoxanthin level in a 50 μL plasma–derived sample using HPLC, we could not observe a peak, as shown in **Fig 2B**. In addition, there were no peaks that were estimated to correspond to neochrome that were derived from neoxanthin detection.

### Plasma β–carotene and lutein concentration

The plasma β–carotene and lutein concentrations significantly increased over the 28 days of young spinach leaf ingestion (Table 1). This degree of increase was approximately 1.4– and 1.9–fold for β–carotene and lutein, respectively.

**Table 1:** Plasma levels of β–carotene and lutein. Plasma samples obtained from participants (n = 14) before (Day 0) and after (Day 28) ingesting young spinach leaves. Data represent means ± standard deviation.

	Before (Day 0)	After (Day 28)	*P* value ^a^
**β–carotene**	0.47 ± 0.20	0.84 ± 0.35	0.0016
**Lutein**	0.34 ± 0.10	0.74 ± 0.28	< 0.001

^a^ *P* values were calculated by Student’s *t*–test.

## Discussion

In this study, we evaluated the bioavailability of neoxanthin when the young spinach leaves containing neoxanthin were ingested without heating. Furthermore, by designing the study to be able to detect and measure the neoxanthin levels with greater accuracy than in a previous study by Asai *et al*. [20], we re–evaluated their conclusion that the bioavailability of neoxanthin is low. We detected neither neoxanthin nor neochrome in the human blood samples. This was a result that further ruled out the possibility that neoxanthin is used in the body, whereas Asai *et al*. [20] were still able to detect them in human blood.

We will discuss the reasons underlying our results by comparing the present study with that by Asai *et al*. [20] (hereinafter referred as to “the previous study” in this paragraph unless otherwise specified) from several points of view. First of all, we perceived that the present study would be more advantageous for detecting neoxanthin than the previous study for the following reasons: 1) only 5 participants were enrolled in the previous study (all adult women), whereas 14 (adult men and women) participants were enrolled in ours; 2) the intake period in the present study was 4 weeks (28 days), as opposed to one week (7 days), in the previous study; 3) the neoxanthin intake in the present study was 3.5 mg day^–1^, as opposed to 3.0 mg day^–1^ in the previous study; and 4) the limit of detection of neoxanthin in HPLC analysis, as blood neoxanthin concentration, was 0.5 nmol L^–1^ in the present study, as opposed to 1 nmol L^–1^ in the previous study. Many of these points provided conditions for the present study to detect neoxanthin with greater accuracy. On the other hand, one possible disadvantage of the present study compared to the previous study was the form of the spinach that was eaten. In the previous study, the participants ate mature spinach leaves that were boiled, cut into 5 cm pieces, and sautéed with olive oil and salt. In our study, participants were asked to eat the young spinach leaves in their raw form, accompanied by some salad dressing. It has been reported that these differences can affect the absorption of carotenoids [24–26]. For example, it has been reported that lipophilic carotenoids are absorbed more when ingested with oil [27, 28]. In this sense, the previous study used olive oil (approximately 12 g day^–1^ of lipids), while in the present study we drizzled the young spinach leaves with salad dressing (approximately 21 g day^–1^ of lipids; assuming that 100 g of young spinach leaves were drizzled with approximately 60 g of salad dressing and making the calculations based on the mean lipid content of the three types of salad dressing used). Therefore, it is unlikely that this worked as a disadvantage. Elsewhere, it has been reported that heating and processing breaks down the cell walls in vegetables and increases carotenoid absorption [24, 29, 30]. Asai *et al*. [20] carefully considered this point and used the sautéed spinach mentioned above as a test food. Although this may be a mere speculation because the data on the neoxanthin content in the fresh spinach used in the previous study remain unclear, perhaps the cooking processing reduced the neoxanthin content, but ultimately 3.0 mg of neoxanthin remained in one serving of sautéed spinach. On the other hand, while the participants in this study were instructed to chew the young spinach leaves well while eating, this may not have broken down the cell walls as much as heating and cooking does. Although eating neoxanthin–containing vegetables in their raw form (without heating) may be effective for preventing a reduction of the neoxanthin levels [15] in the vegetables, it may be a disadvantage from the viewpoint of absorption. In this study, the neoxanthin intake was more than 10% higher per day than in the previous study but we speculate that this was overcome by the improvement in neoxanthin absorption through cooking and processing.

According to a review by Kotake-Nara and Nagao [21], on the multiple animal experiments evaluating the absorption of various carotenoids, when the experiments were conducted under the same conditions, the carotenoid concentrations in mouse plasma 2 h after administration were 36 nM (β–carotene), 10 nM (lutein), 35 nM (neoxanthin and neochrome), and 50 nM (fucoxanthinol and amarouciaxanthin A), and the neoxanthin absorption was around the same level as that of β–carotene and lutein. Thus, at least in mice, the selective absorption of carotenoids has not been observed. On the other hand, it has been suggested that in humans, unlike in mice, the absorption of epoxy group–containing xanthophylls, such as neoxanthin, is low [31, 32]. To be specific, as a result of having human participants ingest paprika oleoresin containing violaxanthin, which has an epoxy group, and by measuring the carotenoid concentration in chylomicrons after ingestion, epoxide group–containing xanthophylls such as violaxanthin and capsanthin 5,6–epoxide were not detected and, in addition to 9–*cis* zeaxanthin, whose level was lower in oleoresin than that of the aforementioned epoxide–containing xanthophylls, zeaxanthin, β–cryptoxanthin, and β–carotene were detected [31]. In another report, violaxanthin and any metabolites thereof were not detected in the blood of humans who ingested corn oil in which violaxanthin was suspended [32]. The above suggests that the bioavailability of epoxy group-containing xanthophylls such as neoxanthin differs significantly between animals and humans. The possible reasons are discussed process-by-process. First, we focus on the process by which dietary carotenoids are solubilized into mixed micelles consisting of bile acids, phospholipids, cholesterol, fatty acids, and monoacylglycerols during the digestive process, and these mixed micelles are taken up by intestinal epithelial cells. Sugawara *et al*. [33] evaluated the uptake of various carotenoids solubilized in mixed micelles into Caco-2 cells (a commonly used human small intestinal cell model) and reported a correlation between the amount of uptake and the lipophilicity of the carotenoids. Of these, the solubility of carotenoids into mixed micelles (i.e. bioaccessibility) has been reported to be more favorable for more polar carotenoids including neoxanthin than less polar ones [29, 34]. On the other hand, it has also been reported that dietary fiber and divalent minerals derived from the food matrices can inhibit the solubilization of carotenoids [35, 36]. Although, in the present study, raw young spinach might have more fiber and minerals than boiled spinach in the previous study [20], there is no data to our knowledge that highly polar carotenoids are more inhibitory. Regarding the uptake of carotenoids from mixed micelles into small intestinal epithelium, Kotake *et al*. [37] revealed in detail that the constituents of mixed micelles affect the carotenoids uptake into Caco-2 cells by regulating ease of carotenoid release. In mixed micelles containing phosphatidylcholine, and digalactosyldiacylglycerol and sulfoquinovosyldiacylglycerol which are also abundant in leafy vegetables, the carotenoids form aggregates or multimes [38, 39], and therefore are difficult to be released from the mixed micelles and be incorporated by Caco-2 cells [40]. Second is the mechanism of absorption in small intestinal epithelium. It has been suggested that the absorption of carotenoids is mediated by membrane protein-mediated facilitated diffusion in addition to simple diffusion [21]. Specifically, the involvement of SR-B1 (Scavenger Receptor Class B Member 1), ATP-binding cassette (ABC) transporters, ABCG5 and ABCG8 (via the excretion of plant sterols in the intestinal tract) has been discussed. Neoxanthin, an epoxy-containing xanthophyll, is considered to be disadvantageous for absorption by simple diffusion due to its polarity. In addition, it has been suggested that neoxanthin has a higher affinity for MDR, a known efflux pump for fat-soluble compounds, than other carotenoids [41]. However, this point is still inconclusive, as contradictory results have been obtained in cells of mouse and human origin [21]. Third, regarding metabolism, neoxanthin is known to be metabolized to neochrome during digestion, but there is little information on the presence and dynamics of other metabolites in human tissues [21]. The fourth concerns the accumulative nature of neoxanthin. Although there is little report on neoxanthin, fucoxanthin, which has a similar structure to neoxanthin, has been reported to have a half-life of only 7 hours (as fucoxanthinol) in human blood [42]. Considering that the blood half-life of other xanthophylls such as lutein and zeaxanthin is at least several days [43], the excretion of fucoxanthin from human tissues is extremely quick, and it would be as difficult to detect neoxanthin in blood as fucoxanthin. If the detail reasons of neoxanthin’s low bioavailability are clarified in the future, it is expected that it will lead to the development of a method for increasing the absorption efficiency of neoxanthin, which has high bioactivity *in vitro*.

One of the limitations of this study is that neoxanthin–derived neochrome was not sufficiently captured during the digestive process. According to a report by Asai *et al*. [19] on the conversion of neoxanthin to neochrome, a total of 24% of neoxanthin and neochrome remained when neoxanthin was used in a digestive juice tolerance test using an artificial digestive juice model. Considering that the residual rates of β–carotene and lutein used in the same test were 5% and 20%, respectively, the total residual levels of neoxanthin and neochrome were not low, and it is unlikely that decomposition during the digestive process could not be detected in the blood. Therefore, as mentioned above, it was considered that although neoxanthin withstood digestion as neoxanthin or neochrome, these compounds were not absorbed by the small intestine. Moreover, a strict balance test was not conducted as part of this study. Therefore, it is unclear how the ingested neoxanthin was metabolized, distributed, and excreted. It may have been excreted through the feces without being absorbed by the intestinal tract, or it may have been metabolized by the intestinal microbiota. Alternatively, we cannot rule out that it may be in the blood in a completely different form from that of neoxanthin or neochrome, or be largely localized in erythrocytes not but plasma because of xanthophyll’s property [44]. However, in any case, this study suggests that the fat accumulation inhibitory effect of neoxanthin confirmed *in vitro* is extremely unlikely to occur in humans who have ingested neoxanthin.

In conclusion, although neoxanthin exerts high bioactivity (inhibitory effect on fat accumulation, among others) *in vitro*, its bioavailability in humans is extremely low. We hypothesized that ingesting leafy vegetables high in neoxanthin in their raw form (unheated) might be beneficial because of the heat–instability of neoxanthin, but this study suggests that eating them in their raw form can be a disadvantage with respect to neoxanthin bioavailability.
